# Peripheral cemento-ossifying fibroma of maxilla

**DOI:** 10.4103/0972-124X.75915

**Published:** 2010

**Authors:** Anirban Chatterjee, Neha Ajmera, Amit Singh

**Affiliations:** *Department of Periodontics, Institute of Dental Sciences, Bareilly, Uttar Pradesh, India*

**Keywords:** Fibroma, gingival overgrowth, peripheral cemento-ossifying fibroma

## Abstract

Peripheral cemento-ossifying fibroma is a reactive gingival overgrowth occurring frequently in anterior maxilla. It is a slow-growing benign tumor which may lead to pathologic migration and other periodontal problems, so it should be excised as soon as possible. The recurrence rate of peripheral cemento-ossifying fibroma is reported to be 8% to 20%, so a close postoperative follow-up is required. Herein, we are reporting a similar case of peripheral cemento-ossifying fibroma in the maxillary anterior region.

## INTRODUCTION

Ossifying fibroma is a benign neoplasm arising in craniofacial bones, composed of proliferating fibroblasts with osseous products that include bone and ovoid calcifications; these lesions are well demarcated from the adjacent bone.[1]

There are two types of ossifying fibromas: the central type and the peripheral type. The central type arises from the endosteum or the periodontal ligament adjacent to the root apex and causes expansion of medullary cavity. The peripheral type occurs solely on the soft tissues covering the tooth-bearing areas of the jaws.[2]

In 1872, Menzel first described ossifying fibroma; but only in 1927, Montgomery assigned a terminology to it.[3]

It occurs exclusively on the gingiva and accounts for 3.1%[4] of all oral tumors and for 9.6% of gingival lesions.[5] The pathogenesis of this tumor is uncertain. Due to their clinical and histopathological similarities, some peripheral cemento-ossifying fibromas are believed to develop initially as a pyogenic granuloma that undergoes fibrous maturation and subsequent calcification. It is frequently associated with irritant agents such as calculus, bacterial plaque, orthodontic appliances, ill-adapted crowns and irregular restorations. The mineralized product probably originates from periosteal cells or from the periodontal ligament.[6]

Ossifying fibroid epulis; peripheral fibroma with calcification; peripheral cemento-ossifying fibroma; and calcifying fibroma are the terms which refer to peripheral ossifying fibroma.[3]

Peripheral cemento-ossifying fibroma appears as a nodular mass, either pedunculated or sessile. It most commonly appears to originate from interdental papilla. The color ranges from red to pink, and the surface is frequently but not always ulcerated. It is more commonly seen in the first and second decades of life and has a female preponderance. There is a slight predilection for the maxillary arch (60%) and the incisor cuspid region (50%).[7] A potential of tooth migration due to the presence of peripheral cemento-ossifying fibroma has been reported.[6] The treatment of choice is surgical excision with removal of irritation factors.

## CASE REPORT

A 45-year-old woman with the chief complaint of painless swelling in upper right front region reported to the Department of Periodontics, Institute of Dental Sciences, Bareilly. She reported that the lesion was present for the last 7 months. It was of pea size when it started, and gradually it increased to attain the present size.

**Figure 1:**
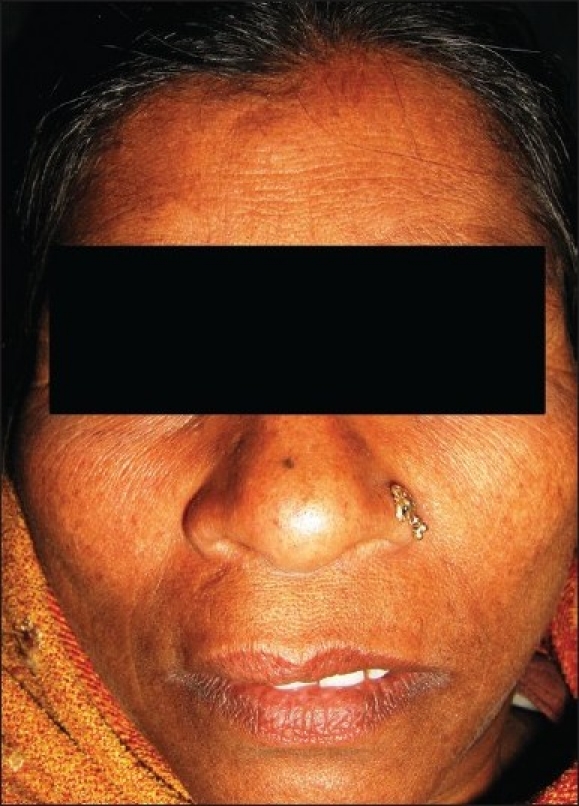
Photograph showing extraoral swelling on right front region, extending from philtrum to angle of mouth

On extraoral examination, swelling was present on the right side, extending from philtrum to angle of mouth [Figure 1].

Intraoral examination revealed a gingival overgrowth in relation to interdental papilla between 11 and 12 of approximately 1×1.5 cm in size, pedunculated, with color same as that of adjacent gingiva. Surface was smooth with few areas of ulceration.

**Figure 2:**
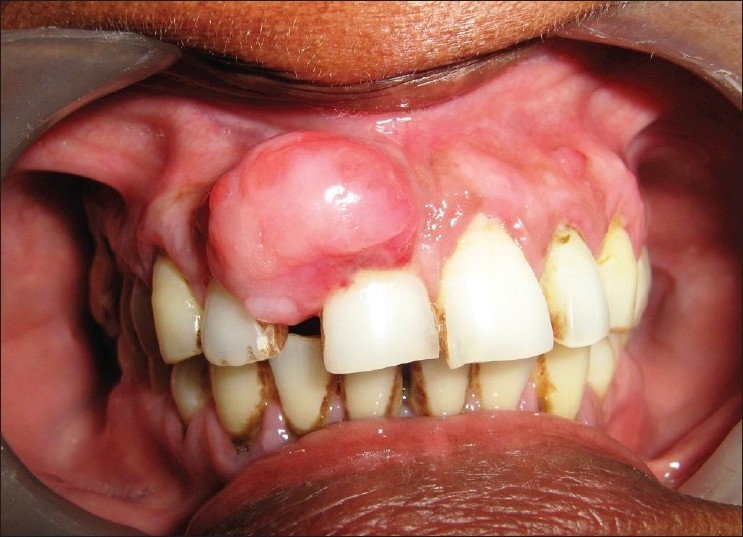
Photograph showing gingival overgrowth extending from right central region to lateral incisor region

Teeth associated were grade II mobile and pathologically migrated [Figure 2].

**Figure 3:**
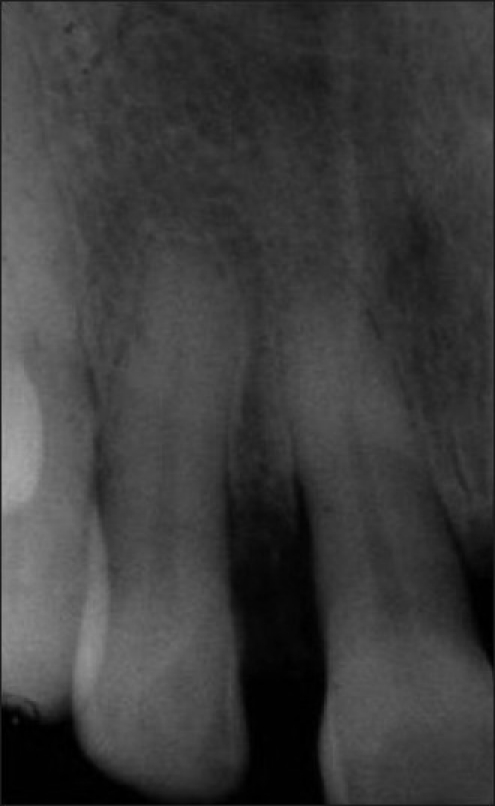
Intra oral Peri Apical showing moderate bone loss in 11, 12 region

**Figure 4:**
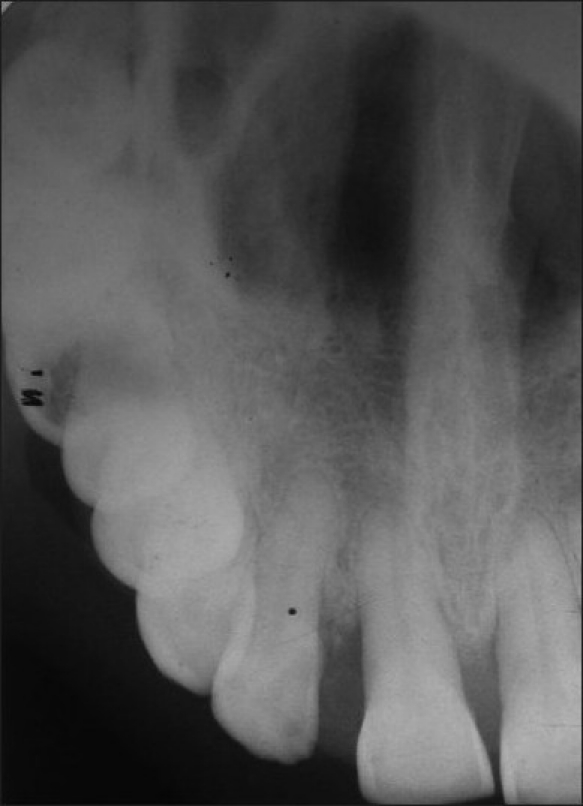
Maxillary occlusal view showing bone loss in 11, 12 region

Radiographic examination revealed moderate amount of bone loss in relation to 11 and 12 in both intraoral periapical and occlusal radiographs [Figures 3 and 4]. Routine hemogram was found to be normal. A provisional diagnosis of peripheral cemento-ossifying fibroma was made. The differential diagnosis included irritational fibroma, pyogenic granuloma and peripheral giant cell granuloma.

**Figure 5:**
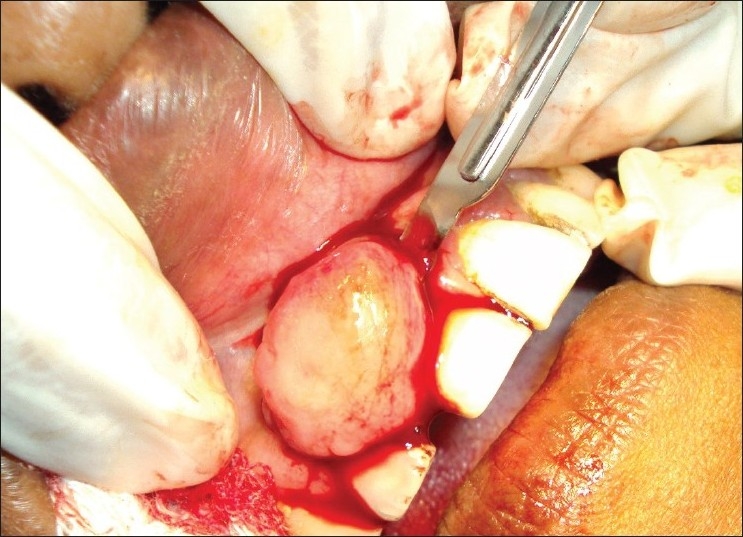
Photograph showing excision of growth

**Figure 6:**
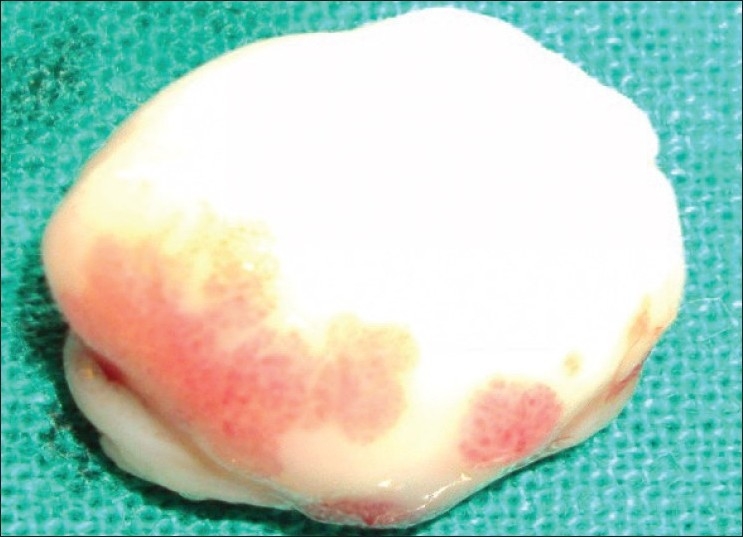
Photograph showing excised tissue

**Figure 7:**
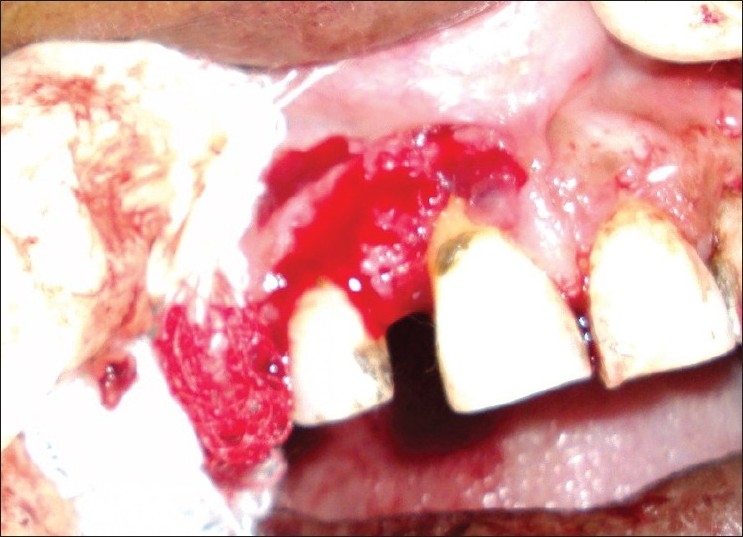
Photograph showing surgical area after excision

The patient had no systemic problem, and surgery was planned on the basis of the clinical and radiographic examinations 7 days after thorough oral prophylaxis. After extraoral and intraoral antisepsis, local anesthesia was given. Excision of the lesion was done, followed by curettage of the area and scaling of the involved teeth. Periodontal dressing was placed [Figures 5–7]. The patient was recalled after 1 week for removal of dressing and checkup.

The tissue excised was sent to the Department of Oral Pathology for histopathological examination.

**Figure 8:**
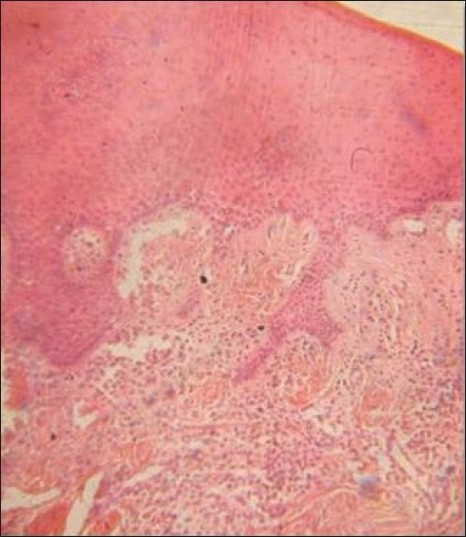
Photograph showing histopathological picture of lesion

Histopathological report revealed parakeratinized epithelium, dense fibrous connective tissue stroma which comprised of plump-to-stellate fibroblasts, along with spindle-shaped fibroblasts. Connective tissue also showed large hematoxyphilic areas of calcification/osteoid with few areas showing entrapped osteocytes and peripheral lining of osteoblast-like cells. Focal areas of inflammation were seen in connective tissue, mainly comprising of lymphocytes and few plasma cells [Figure 8].

**Figure 9:**
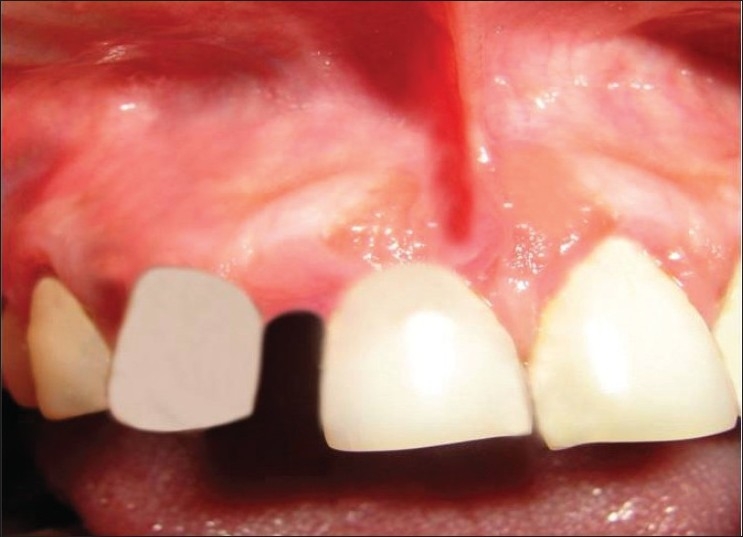
Photograph showing postoperative view at 3 months

The patient was recalled every third month for maintenance therapy and to check for possible recurrence [Figure 9].

## DISCUSSION

Peripheral cemento-ossifying fibroma is a focal, reactive, non-neoplastic tumor-like growth of soft tissue, often arising from the interdental papilla.[8] A lot of confusion has prevailed in the nomenclature of peripheral ossifying fibroma, with various synonyms being used, such as peripheral cementifying fibroma, ossifying fibro-epithelial polyp, peripheral fibroma with osteogenesis, peripheral fibroma with cementogenesis, peripheral fibroma with calcification, calcifying or ossifying fibrous epulis and calcifying fibroblastic granuloma.[9] Ossifying fibromas elaborate bone, cementum and spheroidal calcifications, which has given rise to various terms for these benign fibro-osseous neoplasms. When bone predominates, ‘ossifying’ is the appellation, while the term ‘cementifying’ has been assigned when curvilinear trabeculae or spheroidal calcifications are encountered.[10] When bone and cementum-like tissues are observed, the lesions have been referred to as cemento-ossifying fibroma.

Cementifying fibromas may be clinically and radiographically impossible to separate from ossifying fibromas.[11] An attempt has been made by Endo *et al*. to distinguish cementifying fibromas from ossifying fibromas and fibrous dysplasias by using immunohistochemical analysis for keratan sulfate and chondroitin-4-sulfate, in which the cementifying fibromas showed significant immuno-reactivity for keratan sulfate, and ossifying fibromas and fibrous dysplasias showed intensive immunostaining for chondroitin-4-sulfate.[12]

Though the etiopathogenesis of peripheral ossifying fibroma is uncertain, an origin from cells of the periodontal ligament has been suggested. The reasons for considering periodontal ligament origin for peripheral ossifying fibroma include exclusive occurrence of peripheral ossifying fibroma in the gingiva (interdental papilla), the proximity of gingiva to the periodontal ligament, and the presence of oxytalan fibers within the mineralized matrix of some lesions.[9] Excessive proliferation of mature fibrous connective tissue is a response to gingival injury, gingival irritation, subgingival calculus or a foreign body in the gingival sulcus. Chronic irritation of the periosteal and periodontal membranes causes metaplasia of the connective tissue and resultant initiation of formation of bone or dystrophic calcification. It has been suggested that the lesion may be caused by fibrosis of the granulation tissue.[13]

Lesions involving the gingival soft tissues are rare compared to the lesions appearing within bone.[11] Mesquita (1998) found higher numbers of argyrophilic nucleolar organizer regions (AgNORs) and proliferating cell nuclear antigen (PCNA)-positive cells in ossifying fibroma than in peripheral ossifying fibroma, indicating higher proliferative activity in ossifying fibroma. X-ray diffraction analysis indicated that the mineral phase of both central and peripheral tissues consists of apatite crystals and that the crystallinity of these apatites is lower than that of bone apatite. Also, it was suggested that the crystallinity of the apatites might improve progressively with the development of the lesion, possibly to the same degree as that of bone apatite.[14]

The reported gingival overgrowth has been clearly diagnosed as peripheral cemento-ossifying fibroma after histopathologic examination. Clinical picture of less vascular growth rules out the possibility of pyogenic granuloma.

Histopathology showed no presence of giant cells in connective tissue stroma, thus ruling out the possibility of peripheral giant cell granuloma.

Furthermore, peripheral cemento-ossifying fibroma tends to occur in the second and third decades of life, with peak prevalence between the ages of 10 and 19 years. Almost two thirds of all cases occur in females, with a predilection for the anterior maxilla, i.e., incisor canine region. In the present case, the findings correlate with the general characteristics except for age. The size of the peripheral ossifying fibroma ranges from 0.4 to 4.0 cm. In the present case, the dimensions of the lesion were well within the above-mentioned range. Further, the diagnosis was confirmed by histopathologic evaluation.

Lesion was successfully treated, and follow-up was done at 8 months to check for any recurrence.

Peripheral cemento-ossifying fibroma is a slowly progressing lesion, with limited growth. Close postoperative follow-up is required because of the growth potential of incompletely removed lesions, as well as 8% to 20% recurrence rate.[8] It is important to remove lesions completely by including subjacent periosteum and periodontal ligament, besides the possible causes, to reduce recurrence.
